# User involvement in a Cochrane systematic review: using structured methods to enhance the clinical relevance, usefulness and usability of a systematic review update

**DOI:** 10.1186/s13643-015-0023-5

**Published:** 2015-04-20

**Authors:** Alex Pollock, Pauline Campbell, Gillian Baer, Pei Ling Choo, Jacqui Morris, Anne Forster

**Affiliations:** Nursing, Midwifery and Allied Health Professions Research Unit (NMAHP RU), Glasgow Caledonian University, Cowcaddens Road, Glasgow, G4 0BA UK; Department of Physiotherapy, Queen Margaret University, Queen Margaret Drive, Edinburgh, EH21 6UU UK; School of Health and Life Sciences, Glasgow Caledonian University, Cowcaddens Roads, Glasgow, G4 0BA UK; School of Nursing and Midwifery, University of Dundee, 11 Airlie Place, Dundee, DD1 4HJ UK; Academic Unit of Elderly Care and Rehabilitation, Bradford Institute for Health Research, Bradford Teaching Hospitals NHS Foundation Trust/University of Leeds, Duckworth Lane, Bradford, BD9 6RJ UK

**Keywords:** Cochrane review, User involvement, Stakeholders, Consensus methods, Nominal group technique, Physiotherapy, Stroke

## Abstract

**Background:**

This paper describes the structured methods used to involve patients, carers and health professionals in an update of a Cochrane systematic review relating to physiotherapy after stroke and explores the perceived impact of involvement.

**Methods:**

We sought funding and ethical approval for our user involvement. We recruited a stakeholder group comprising stroke survivors, carers, physiotherapists and educators and held three pre-planned meetings during the course of updating a Cochrane systematic review. Within these meetings, we used formal group consensus methods, based on nominal group techniques, to reach consensus decisions on key issues relating to the structure and methods of the review.

**Results:**

The stakeholder group comprised 13 people, including stroke survivors, carers and physiotherapists with a range of different experience, and either 12 or 13 participated in each meeting. At meeting 1, there was consensus that methods of categorising interventions that were used in the original Cochrane review were no longer appropriate or clinically relevant (11/13 participants disagreed or strongly disagreed with previous categories) and that international trials (which had not fitted into the original method of categorisation) ought to be included within the review (12/12 participants agreed or strongly agreed these should be included). At meeting 2, the group members reached consensus over 27 clearly defined treatment components, which were to be used to categorise interventions within the review (12/12 agreed or strongly agreed), and at meeting 3, they agreed on the key messages emerging from the completed review. All participants strongly agreed that the views of the group impacted on the review update, that the review benefited from the involvement of the stakeholder group, and that they believed other Cochrane reviews would benefit from the involvement of similar stakeholder groups.

**Conclusions:**

We involved a stakeholder group in the update of a Cochrane systematic review, using clearly described structured methods to reach consensus decisions. The involvement of stakeholders impacted substantially on the review, with the inclusion of international studies, and changes to classification of treatments, comparisons and subgroup comparisons explored within the meta-analysis. We argue that the structured approach which we adopted has implications for other systematic reviews.

**Electronic supplementary material:**

The online version of this article (doi:10.1186/s13643-015-0023-5) contains supplementary material, which is available to authorized users.

## Background

The act of professionals and consumers^a^ working together to produce and share knowledge has been an explicit principal of the Cochrane Collaboration since it first began in 1993 [1,2]. A variety of rationales have been proposed to support this active involvement of people with a health condition in systematic reviews, but the principal arguments are that this involvement is beneficial to the quality, relevance and impact of health research [3,4]. The active involvement of people with a health condition has been proposed as a way to enhance the perceived usefulness of systematic review evidence, addressing barriers to the uptake of synthesised research evidence [5]. However, despite widespread acceptance of these arguments, which are driving national strategies to ensure involvement of people affected by health conditions in all research activities [6], there remains a lack of high-quality evidence demonstrating the impact of involvement on research activity and uptake of evidence [3]. Arguably, this lack of evidence has contributed to the considerable inconsistencies in the extent of user involvement within Cochrane reviews, despite over 20 years of consumer involvement within the Cochrane Collaboration [7].

Where there has been user involvement^a^ within individual Cochrane reviews, the approaches to involvement have also varied considerably [7]. A review of user involvement in systematic reviews confirmed that a wide range of different approaches to involvement has been implemented [8]. This review found that the most commonly used approaches are consultation with a group of people at a one-off workshop or at key stages in the review process, or involvement of individual people as members of a review team, although other approaches such as email consultation and using a Delphi process have also been used [8].

Challenges to the development of effective methods of involvement within individual reviews are compounded by poor description of involvement within many reviews [8] and by limited evaluation of the impact of involvement [3]. However, based on an exploration of case examples, Boote proposes a number of strategies to facilitate effective involvement within systematic reviews [8]. These include budgeting for the costs of involvement; having a review team member with lead responsibility for involvement; providing training, briefing notes and background information to people involved; and the use of structured methods of involvement (such as the nominal group technique or Delphi process) at key stages of the review process.

In order to enhance the clinical relevance of a Cochrane systematic review relating to physiotherapy after stroke, we aimed to use structured methods, based on Boote’s suggested strategies [8], to involve patients, carers and health professionals in an update of this review.

### Cochrane systematic review

Stroke is a leading cause of death and disability and is within the top ten causes of long-term physical disability in many Western nations [9-11]. The most common and widely recognised impairment caused by stroke is motor impairment, with paralysis of some parts of the body, difficulties with various physical functions and limitations in mobility [12]. Consequently, the ultimate goal of physical rehabilitation is most commonly the improvement of function and mobility [13]. Over the years, various approaches to physical rehabilitation have been developed, according to different ideas about how people recover after a stroke. Considerable debate continues among physiotherapists^b^ about the relative benefits of different approaches [14,15].

Our Cochrane systematic review explored whether physical rehabilitation approaches are effective in recovery of function and mobility in people with stroke and if any one physical rehabilitation approach was more effective than any other approach. It was first published in 2003 [16] and updated in 2007 [17]. However, within the 2007 version, a number of limitations were identified, and the review authors recommended that these should be addressed within any subsequent updates [17]. These limitations related to the methods of defining and categorising physical rehabilitation approaches within the review, which were based on historical development of approaches within Western nations. The first concern was that the methods used within the review to define and categorise interventions were no longer relevant to current clinical practice. The second concern was whether physical rehabilitation interventions described in foreign-language publications (many of which were listed as ‘awaiting assessment’ in the 2007 version) were relevant for inclusion and - if they were - how these international approaches could best be categorised within the review.

**Figure 1:**
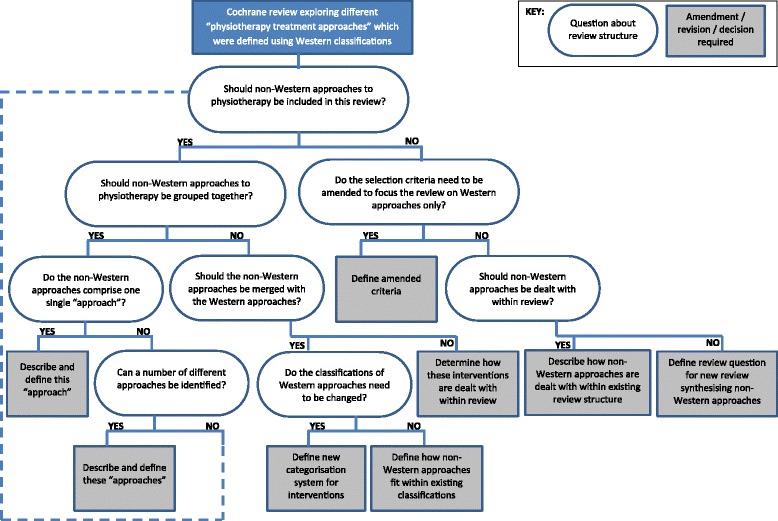
**Possible amendments to Cochrane review identified within project plan.**

Prior to the next update [18], we therefore planned to amend the structure and format (without expanding the scope) of this review in order to address these limitations and produce a review which had international clinical relevance. We planned to explore the approaches described in the foreign-language publications and clarify whether it was clinically relevant and useful to synthesise evidence relating to these international approaches within this review. While it was clear from the 2007 version that amendments were required to address limitations within the review, the nature of these amendments was not clear. There were therefore a number of important decisions to be made which could substantially affect the structure, scope and methods of the review. These decisions would significantly impact on how the evidence was synthesised within the updated review, with potential solutions varying from amending the classification system with the existing review to having two separate reviews. Key decisions and potential solutions are illustrated in Figure 1. We sought to address these issues and make decisions through involvement of a group of stroke survivors, carers and physiotherapists and educators (‘stakeholder group’) in the review update.

This paper describes the structured methods of involvement of this stakeholder group in the updated Cochrane review and explores the perceived impact of involvement. The methods and results of the review have been fully presented within the Cochrane Library [18].

## Methods

**Figure 2:**
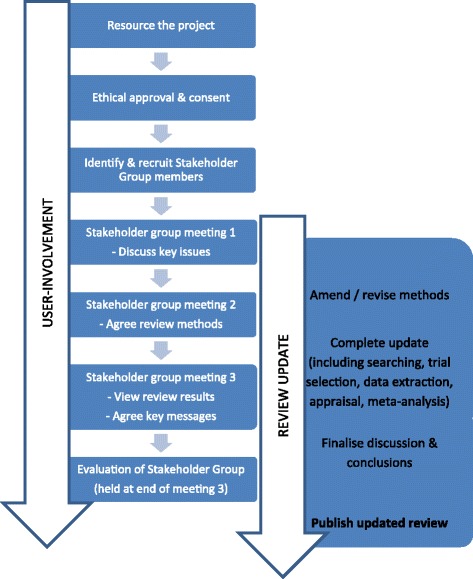
**Overview of structured methods of involvement during review update.**

Figure 2 provides an overview of the key stages of the structured methods of involvement in relation to the updating of the Cochrane review.

### Resourcing the project

We sought funding to support the stakeholder group involvement in this project. We costed for the direct expenses associated with involvement (that is, travel, subsistence), but not for any funding to pay for the time of group members. This decision was based on our prior experience of working with stroke survivors and carers, where we found that people were generally happy to be involved in stroke research without compensation for their time. Furthermore, we considered that the involvement of physiotherapists and educators ought to provide these individuals with an opportunity for professional development and be carried out during their employed working hours (through negotiation with relevant managers). We costed for a part-time researcher (for 12 months) who would play the lead role in management of the stakeholder group, as well as contribute to the tasks involved in updating the review (including running searches, identifying and appraising trials, extracting and entering data).

### Ethical approval and consent

There has been some debate relating to the necessity to gain ethical approval for the involvement of people within the process of healthcare research (that is, as opposed to participants in research) [8,19]. However, as we planned to audio-record group meetings and use this qualitative data as a record of group discussion and decisions, we did seek ethical approval for the stakeholder group involvement within this project (from Glasgow Caledonian University School of Health and Life Sciences ethics committee). We gained signed informed consent from all group participants, specifically relating to the collection of audio data.

### Identifying and recruiting stakeholder group members

We planned to recruit a stakeholder group comprising stroke survivors, carers, physiotherapists and educators. As this review was focussed on interventions that are generally exclusively delivered by physiotherapists, we decided that physiotherapists should be the only health professionals involved. We planned to purposefully select physiotherapists to ensure a variety of grades, years of experience, post-graduate courses and geographical work base. We planned to recruit between 8 and 12 group members as previous studies using the nominal group technique have recommended between 5 and 9 participants [20], and we predicted that some group members may not attend all meetings so we wished to over-recruit.

We formed a two-page role description [see Additional file 1], which detailed the aim and purpose of the stakeholder group, what was required in terms of skills, experience and knowledge and the time commitment (including the dates of all meetings), what the potential benefits of involvement could be and confirmation of re-imbursement of any travel expenses. Separate versions were written for stroke survivors/carers and physiotherapists/educators. We circulated this description, with a request for interested people to contact us via email using established professional, charity and patient-support networks across Scotland. We limited recruitment to people living in Scotland due to the requirement to attend meetings within Central Scotland and our limited available travel budget. Physiotherapists who responded were asked to complete a simple questionnaire to provide details of their current position, clinical experience and relevant post-graduate training. This information was used to select physiotherapists for inclusion within the group, according to our sampling strategy, in order to give representation of the criteria listed above.

### Stakeholder group meetings

We pre-planned three stakeholder group meetings during the course of the 12-month project. The content, structure and format of these three meetings, and details of the statements which were discussed and voted on, are illustrated in a table [see Additional file 2]. Meetings 1 and 2 were held at the start of the project, prior to the review update being carried out. The objectives of meeting 1 were to explore and reach consensus on how physical rehabilitation approaches should be categorised, including decisions over whether international approaches should be included, and - if so - how these should be categorised within the review. Discussions were focused around pre-determined statements on which consensus decisions were desired. The objectives for meeting 2 were to explore and agree on specific strategies to update and amend the current Cochrane systematic review, based on the decisions made during meeting 1. Meeting 3 was held at the end of the project when the results of the review update were complete, and the objective of this meeting was to explore the perceived clinical implications of the findings. During meeting 1, the stakeholder group also briefly debated how the results of the review should be disseminated to NHS Scotland practitioners and how evidence-based practice could be encouraged, and an action plan for dissemination activities was drafted. This dissemination plan was revisited in meetings 2 and 3, and plans for local dissemination across NHS Scotland discussed and agreed.

Meeting ‘ground rules’ were discussed and agreed by all group members at the start of each meeting. These included turning off mobile phones and electronic devices, raising a hand when wanting to speak, no person speaking for more than 2 min at any one time, respecting each other’s opinions and not talking over other people. Each meeting had a designated facilitator and timekeeper. We sent relevant background papers prior to each meeting, and researchers provided brief presentations of key material at the start of each meeting.

### Consensus methods

**Figure 3:**
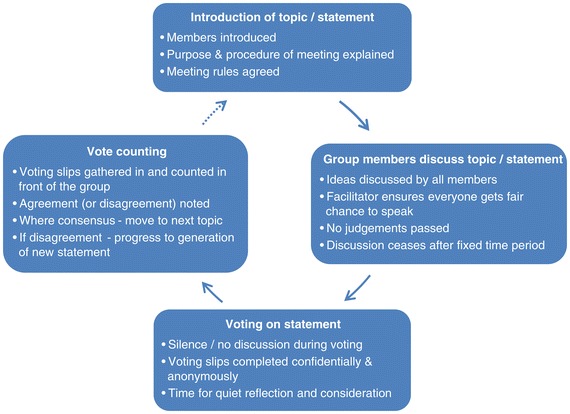
**Illustration of key stages of nominal group technique.**

We used formal group consensus methods to reach consensus decisions within the stakeholder group meetings, as such methods are recognised to be advantageous when subjective judgements need to be organised [21]. These consensus methods were based on nominal group techniques (see Figure 3), as this method enables the pooling of decisions and judgements from a group of informed experts, leading to votes on a range of options until ultimately group consensus is reached [20,22]. The structure of each meeting involved a set time period for discussion around a statement or issue, followed by voting. The statements for meeting 1 were pre-determined by the research team, while statements for subsequent meetings evolved and were agreed through group discussion. No group discussion occurred during the voting, which was completed anonymously on a paper slip. For the statement which was discussed, each individual member ranked their agreement with the statement on a scale of 1 (strongly agree) to 5 (strongly disagree) and provided written comments on their reason if they wished [see Additional file 3 for a sample voting slip]. Immediately after voting, the paper slips were gathered in, counted and presented to the group. If there was a lack of consensus in the responses, we planned further discussion around a second statement (the wording of which would have been agreed by the group) and subsequent rounds of voting as required; however, no second rounds of discussion or voting were required during either of the meetings. Written comments were tabulated, with reference to the score maintained.

Some of the review authors attended the stakeholder group meetings and contributed to discussions, as it was felt their attendance was important in order to answer any questions that the stakeholder group may have relating to the existing review and Cochrane review methods. However, review authors did not participate in the voting process.

Analysis involved determining the proportion of respondents agreeing with each statement within each round of voting. In addition, consensus decision meetings were audio-recorded and transcribed verbatim to facilitate further analysis of qualitative data relating to the themes discussed around specific statements.

### Additional contact with stakeholder group members

In addition to the three planned meetings, contact was made with stakeholder group members by email as required throughout the project. Researchers contacted members to seek feedback or comments on specific issues relevant to the stage of the review update, and a variety of methods were used to gain feedback. Additional file 4 provides an overview of the range of methods used to gain feedback and involvement. These methods included the use of a number of feedback forms and the use of voting around specific issues or decisions (using Doodle polls).

### Evaluation of stakeholder group

At the end of meeting 3, we circulated a brief evaluation form requesting written feedback on the process of involvement and the perceived impact of involvement. We also had an informal group discussion around the perceived impact of involvement, which was audio-recorded and transcribed.

## Results

We gained funding to support this project from the Scottish Government’s Health Directorate, Chief Scientist Office (ref: CZG/2/544). The project was approved by the Glasgow Caledonian University School of Health and Life Sciences ethics committee (Reference: HLS12/40).

Thirteen people were recruited onto the stakeholder group. This comprised three stroke survivors, one carer and nine physiotherapists. Only four stroke survivors/carers volunteered to participate; all four joined the group. The nine physiotherapists represented a wide range of clinical experience, geographical locations and post-graduate experience. An additional four physiotherapy educators/researchers, who were all authors on the Cochrane review, attended and contributed to the stakeholder group meetings in order to clarify issues (when required) during discussion but were excluded from the voting process.

### Meeting 1

The results of the voting in meeting 1 are presented in Table 1. There was generally a consensus that the categories which had been used in the 2007 version of the review were no longer appropriate or clinically relevant (statement A). There was unanimous agreement that the international trials (which had not fitted into the previous method of categorisation) ought to be included within the review (statement B). Discussion highlighted the need to ensure that the review did not exclude evidence based on geographical boundaries or norms of practice. Participants recognised challenges relating to clear descriptions of the physical rehabilitation approaches and achieving clinically relevant and useful categorisations of these. There was less clear agreement regarding the similarity of content of the physical rehabilitation approaches investigated by the international trials which had been presented (statement C), but discussion and comments clearly highlighted a common perception of some similarities between the interventions described in the trials.

**Table 1:** **Results of voting at stakeholder group meeting 1**

	**Total number of responses**	**Strongly agree**	**Agree**	**Neither agree or disagree**	**Disagree**	**Strongly disagree**
		**1**	**2**	**3**	**4**	**5**
*Statement A.* The current categories are appropriate and clinically relevant	*n* = 13^a^	0	1	1	9	2
	%100		8	8	69	15
*Statement B.* These international trials should be included in our review of physiotherapy treatment approaches	*n* = 12^a^	6	6	0	0	0
	%100	50	50			
*Statement C.* The interventions studied in these trials are similar to one another	*n* = 12^a^	2	7	3	0	0
	%100	17	58	25		

^a^Thirteen participants attended the first half of meeting 1 and voted on statement A. One participant then had to leave the meeting, and the remaining 12 participants voted on statements B and C.

Discussion at the end of meeting 1 confirmed the potential benefits of defining ‘treatment components’, and at the end of this meeting, the group members asked the researchers to complete additional tasks. This involved the systematic exploration of the treatment components reported within the intervention descriptions of all the trials that had been included within the 2007 version of the review. This led to the production of materials which were sent out to meeting participants prior to meeting 2 [see Additional file 2 for more details].

In addition, although the stakeholder group members were not specifically asked to debate this, the importance of exploring particular subgroups came up repeatedly within the discussion. For example, discussion emphasised the importance of the amount (or ‘dose’) of intervention provided and the stage of the rehabilitation process of participants. These discussions enabled the researchers to identify subgroups which were perceived to be important by the group and to subsequently amend the protocol to reflect these agreed subgroups.

### Meeting 2

Prior to meeting 2, the stakeholder group members were sent descriptions of the treatment components from the 30 trials, which were either included or awaiting assessment in the 2007 version of the review. Group members were asked to consider and send feedback relating to how the interventions within individual studies could be categorised and how the treatment components described as part of these interventions might be grouped. We received feedback from ten individual people, which was collated, and presented (anonymously) to the whole group at the start of meeting 2. This presentation clearly highlighted where there had been agreement between responses and where there had been disagreement. Following these presentations, the group members were asked to decide how to progress toward reaching agreement over the categorisation and definition of treatment components within the interventions.

Group members decided to work together to produce agreed descriptions and definitions of the individual treatment components which make up different approaches to physical rehabilitation and to reach agreement on how to categorise these individual treatment components. Through a process of group discussion, 27 individual treatment components were defined and grouped under 7 categories [see Additional file 5]. The wording of each of these definitions was agreed through group discussion, with the researcher typing these into a projected spreadsheet during the discussion. Voting was carried out after all the definitions and categories had been verbally agreed, to ensure that there was consensus over these. This voting demonstrated unanimous agreement with the new categories, and the names given to these categories (see Table 2).

**Table 2:** **Results of voting at stakeholder group meeting 2**

	**Total number of responses**	**Strongly agree**	**Agree**	**Neither agree or disagree**	**Disagree**	**Strongly disagree**
		**1**	**2**	**3**	**4**	**5**
*Statement A.* The new categories are appropriate and clinically relevant	*n* = 12^a^	2	10	0	0	0
	%100	17	83			
*Statement B.* The stated names are appropriate and clinically relevant	*n* = 12^a^	2	10	0	0	0
	%100	17	83			

^a^Twelve participants attended and voted during meeting 2.

### Meeting 3

At meeting 3, the results of the review update were presented by the researchers, with the data grouped according to the treatment components and categories agreed during meeting 2. After the presentation of each meta-analysis within the updated review, the group members debated the clinical implications of the results. At the end of the meeting, the group revisited these clinical implications and discussed and reached agreement on the key messages arising from the review and the implications for physiotherapists, stroke survivors and carers. These agreed key messages were later incorporated into the final version of the written Cochrane review, with acknowledgement that group members had agreed on these during the stakeholder group meeting.

### Additional contact with stakeholder group members

Following meeting 3, contact was maintained with the group members via email. In particular, contact was made in relation to:

- Document summarising Cochrane review findings. A draft document was circulated to group members, and feedback was provided by email.
- Decision to change title of Cochrane review. During the Cochrane peer review process, a suggestion was made that the title ought to be changed (from ‘physical treatment approaches’ to ‘physical rehabilitation approaches’ or to ‘physical therapy approaches’). A Doodle poll was used, enabling all group members to ‘vote’ on the desired title, resulting in clear agreement around a preferred title.
- Collaboration on delivery of a Focussed Symposium at the Annual United Kingdom and Ireland Cochrane Symposium. All group members were asked if they would like to collaborate on a proposal to run a symposium session. Four members volunteered for this, resulting in a successful workshop proposal, which was later delivered by two researchers and four stakeholder group members at the Cochrane Symposium [23].

### Evaluation

Nine group members completed an evaluation form at the end of meeting 3, with all respondents strongly agreeing that the views of the group impacted on the review update, that the review benefitted from the involvement of the stakeholder group and that they believed other Cochrane reviews would benefit from the involvement of similar stakeholder groups. The full results from the written evaluation are provided in Additional file 6. A small number of group members highlighted specific difficulties relating to involvement, including issues around the timing and travel to meetings, and the representativeness of the group members; however, the majority of the feedback was positive and particularly emphasised that group members felt their opinions were valued [‘I feel that the opinions of the stakeholder group were greatly valued’] and that the process of involvement was acceptable [‘Really well organised, structured and productive’] and beneficial to the Cochrane review [‘Other Cochrane groups please copy’]. Furthermore, group discussion clearly emphasised that the level of involvement was perceived to be unique and was highly appreciated:

‘…I have taken part in quite a number of things of this nature over the past 20 years and this is the first time that I have really felt that it has been successful and that I have been listened to..’

‘To have been listened to as clinicians, um, with our aspiration of trying to deliver high quality evidenced-based practice, where there are real challenges doing that, but to really be listened to in that respect and then to translate ……. is pretty unique’

## Discussion

We used clearly described structured methods to involve a stakeholder group in the update of a Cochrane systematic review and collected data which objectively support decision-making within the review process. The involvement of stakeholders impacted substantially on the review, with changes to the inclusion of studies exploring non-Western approaches to physiotherapy, classification of treatments, comparisons and subgroup comparisons explored within meta-analysis and dissemination of key messages. The group members made important decisions around the classifications of interventions within the review, leading to the development of a new method of intervention categorisation and enabling synthesis and analysis of evidence which was perceived to be clinically relevant. The stakeholder group members perceived that their involvement was valuable, and valued, and that the review benefited from their involvement.

We believe that this approach to user involvement has implications for other systematic reviews, and this is supported by the members of the stakeholder group. We have demonstrated that, with appropriate planning and resources, user involvement can be integrated into the process of updating a complex review and timely completion of the review achieved. The use of structured methods, based on the nominal group technique, enabled consensus decisions to be reached in a transparent manner with equal value placed on the opinion of each member of the stakeholder group.

The review that we updated was, arguably, unique in that the previous version had highlighted a number of major limitations which required to be addressed prior to the subsequent update. This meant that there were clear questions for which we were seeking consensus decisions. This, clearly, will not be the case for all review updates. However, during the development of new protocols for new systematic reviews, there are always a number of methodological decisions which have to be made. These decisions include, but are not limited to, the selection criteria for the review (participants, interventions, comparisons and outcomes), planned review comparisons, analyses and subgroup analyses. We believe that user involvement - using structured methods similar to those which we describe - introduced at the stage of protocol development, has the potential to enhance the clinical relevance, usefulness and usability of systematic reviews.

Our review was focussed on highly complex interventions, which comprised a range of treatment components, which could be delivered in different ways, by different professionals in different settings to participants with a wide range of varied impairments and disabilities. These levels of complexity clearly add to the challenges of definition and focus within the review, arguably adding value to the role of stakeholders who bring a range of experiences and opinions. However, even the ‘simplest’ of interventions (for example, a single dose of a drug) will be delivered to participants who will vary, for example, in relation to their disease, medical history, their past experiences and values placed on different outcomes. Consequently, while not explored within this study, we do believe that user involvement within a systematic review has the potential to enhance the clinical relevance, usefulness and usability even when focused on the most ‘simple’ of interventions.

We designed our user involvement based on strategies proposed by Boote [8] to facilitate effective involvement within systematic reviews. We believe that the pre-planning of the project, based on these strategies was a key contributor toward the success of our user involvement. Additional specific techniques that we believe contributed toward successful user involvement included:

- fixing all meeting dates prior to identification and recruitment of stakeholder group members. This ensured that potential recruits were all available for all meetings (with the exception of unforeseeable events such as illness of oneself or a family member)
- agreeing on ‘meeting rules’ which included strategies aimed at ensuring no one person dominated the discussion and that all group members could participate equally (described in the ‘Methods’ section).

Although we pre-planned the user involvement within this project, we did not have any user involvement at the stage of project planning or involvement with funding applications. We acknowledge that this is a limitation of our study and that user involvement at the planning stage may have been beneficial (for example, in relation to decisions about who and how to recruit to the stakeholder group). However, there are many additional challenges associated with user involvement at the project planning stage, often related to the absence of dedicated time and resources at this early stage of project development.

We acknowledge that a key limitation of our user involvement was the composition of our stakeholder group, which comprised four stroke survivors/carers in comparison to nine physiotherapists. While we made attempts to engage greater numbers of stroke survivors/carers, cascading information about the project through a variety of routes, time constraints of the project limited us to electronic dissemination, which clearly restricted the population of people who had the opportunity to volunteer for involvement. We only received responses from four stroke survivors/carers, all of whom joined the group. Ideally, we would have liked to have at least as many, if not more, stroke survivors/carers as health professionals. While there was an imbalance between stroke survivors/carers and health professionals, it is important to note that the physiotherapists involved were ‘users’ of the evidence being synthesised. Although we have no evidence of this, it is possible that our decision not to seek funds to enable us to offer financial support for involvement may have been a barrier to involvement, and we recognise that there is a need for more detailed exploration of the potential barriers and facilitators to involvement. Despite some group members highlighting a sense of dissatisfaction relating to the numbers of stroke survivors/carers, all group members did report that they felt they were listened to and their opinions valued. For future projects in which stakeholder groups are being set up, we recommend that additional time and resources are allocated to the set-up stage to enable greater and more equitable representation of patients and carers.

No members of the stakeholder group became authors on our review, although we did acknowledge the contributions of the individual members within the published review. However, we acknowledge that it is likely to be appropriate within some other reviews to invite members of user groups to contribute as named authors to a review. This would require careful planning to ensure that all review authors had appropriate training and resources to support meaningful contribution and that the roles and responsibilities of individual review authors were clearly defined and agreed at the start of the review process.

Throughout this process, we made it clear to the stakeholder group members that the decisions taken would be the decisions of the group members, and *not* of researchers or review authors. We emphasised the real sense of control that this gave them over the output of the final review. As researchers, we took direction directly from the decisions made by the group members, effectively handing over control to the group in relation to some highly important methodological decisions in relation to the review. At all stages, we strove to be transparent in the reporting of decisions and have ensured that we acknowledged and reported the role of the stakeholder group within the published Cochrane review. Our experience is that the act of effectively ‘handing over control’ felt somewhat ‘alien’ to the researchers, who are generally in the position of having the final say over methodological decisions relating to their research. However, the review authors also found that there was considerable value associated with being able to report ‘implications for practice’ which were reflecting the consensus viewpoint of the stakeholder group, rather than the views of individual researchers or review authors only. This was perceived to add validity to the key messages within the review, consequently contributing to the value placed on the review by patients, carers and health professionals.

The evaluation and feedback from our stakeholder group was highly positive and highlighted that the group perceived their input to have been valued and have a beneficial impact on the final review. However, the participants’ viewpoints are at high risk of bias, and it is a key limitation of our study that we did not have any independent evaluation of the impact of user involvement.

## Conclusions

We have demonstrated that using clear pre-planned structured methods can facilitate effective user involvement within a major update of a complex systematic review. We found that the strategies proposed by Boote were effective, and we identified a number of specific techniques which we believe contributed to successful user involvement [8]. The user involvement led to key decisions relating to the structure and methods of the review, resulting in a review which was perceived to have enhanced relevance, validity and accessibility. We argue that the structured approach which we adopted has implications for other systematic reviews.

## Endnotes

^a^There is no agreed definition of the term ‘consumer’ and lack of agreement that this is the best term to use [2,24]. For the purposes of this paper, we define ‘consumer’ as a person with a healthcare condition, or friend, carer (unpaid) or family member of that person. A discussion of the varied terminology (for example, consumer, public, user) is beyond the scope of this article, and we do not distinguish between these terms. However, within this paper, we have chosen to primarily use the term ‘user involvement’, as we felt that this clearly includes all ‘users’ of health information, including consumers, members of the public and health professionals.

^b^Professionals who deliver physical rehabilitation, also known as physical therapists or rehabilitation therapists.

## Additional files

Additional file 1:
**Role description sent to physiotherapy stakeholder group members.** This is the information sheet which was circulated in order to recruit physiotherapists onto the stakeholder group. Additional file 2:
**Table summarising content and format of stakeholder group meetings.** This table provides details of each of the three stakeholder group meetings, including the statements which were discussed and voted on. Additional file 3:
**Sample voting slip.** This is a copy of one of the voting slips used during stakeholder group meeting 1. The slips were printed on A5 paper. Participants were instructed to circle the appropriate number to show their agreement with the statement and write the reason for their selection in the comments box. Additional file 4:
**Overview of methods used to gain feedback and involvement.** A number of different methods were used to communicate with, and gain feedback and involvement of, the stakeholder group members. This table describes the range of methods used, how these were used and the resulting involvement of the stakeholder group members. Additional file 5:
**Table of individual treatment components, definitions and categories, as defined by the stakeholder group.** This table lists the categories and treatment components as defined by the stakeholder group. These categories and treatment components were used to categorise the interventions of all the trials included within the Cochrane review. Additional file 6:
**Results of written evaluation collected after the third stakeholder group meeting.** Written evaluation was collected from nine participants after the third stakeholder group meeting. Participants ranked their agreement with statements 1 to 8 on a five-point scale from strongly agree to strongly disagree. The first table illustrates the agreement assigned by the nine participants. The feedback form also contained four open-questions, and participants provided written comments in response to these. The second table lists all the written responses (unedited and anonymous) from the nine participants to these four open-questions.
