# Supplementary material for: User involvement in a Cochrane systematic review: using structured methods to enhance the clinical relevance, usefulness and usability of a systematic review update
Source: Syst Rev. 2015 Apr 20;4:55. doi: 10.1186/s13643-015-0023-5 (PMC4407304; doi:10.1186/s13643-015-0023-5)
Supplement: Additional file 1: — Role description sent to physiotherapy stakeholder group members. This is the information sheet which was circulated in order to recruit physiotherapists onto the stakeholder group. [file 13643_2015_23_MOESM1_ESM.pdf]

Stroke Physiotherapy treatment approach Review Update of Cochrane Evidence (SPRUCE project)

We have received funding from CSO to update our existing Cochrane systematic Review of physiotherapy treatment approaches for stroke.[1] This project provides opportunities for physiotherapists to get involved as a member of a 'Stakeholder Group'.

Project Background

This Cochrane review examines the effectiveness of different physiotherapy treatment approaches (e.g. Bobath or motor learning) for key functional, impairment and participation outcomes following a stroke.

Our Cochrane review was last updated in 2007 and concluded that there was insufficient evidence to support the application of any one individual approach. The findings from the review demonstrated that an eclectic approach, involving a mix of components from different approaches, was more effective for improving functional independence. However, since completing this work, a number of potentially relevant studies – including a large number of foreign language publications, not previously included – have been identified. Inclusion of these studies may alter the conclusions of the review and it is important that we update the review.

An up-to-date and comprehensive evidence synthesis will enable appropriate decisions to be made about the use of global physiotherapy approaches for stroke in Scotland. Translation of this knowledge into practice will be essential to foster evidence-base stroke care.

Opportunities to get involved

Central to the success of our study is the engagement of key stakeholders including physiotherapists, NHS managers, clinical educators, and service users. It is our hope that active engagement with key stakeholders in the development, conduct and dissemination of this evidence synthesis will ensure that the final project outcomes are relevant and accessible to staff and patients within Scotland.

[1] Pollock A, Baer G, Pomeroy VM, Langhorne P. Physiotherapy treatment approaches for the recovery of postural control and lower limb function following stroke. Cochrane Database of Systematic Reviews 2007, Issue 1.

We briefly summarised the aims of the project and the level of commitment required from interested stakeholders in the table below.

Aim	To provide guidance and advice to the researchers responsible for updating the 2007 Cochrane Review. This will involve:<br>a. Deciding how to synthesize evidence from non-western approaches within the existing review.<br>b. Agreeing strategies to update/amend the current systematic review.<br>c. Providing guidance on how the findings from the updated review should be disseminated to physiotherapists and other relevant groups<br>d. Identifying any gaps in the evidence base relating to physical rehabilitation approaches for patients with stroke.	
Purpose	A stakeholder group (SG) composed of stroke survivors/service users, carers, physiotherapists, educators and NHS managers/commissioners will be convened to play a key role in establishing specific project output and actions.	
Commitment	Three attendances at NMAHP research unit located within Glasgow Caledonian University.<br>The meetings will take place in the afternoon on the 6th March 2013, the 24th April 2013 and on the 2nd of October.<br>Meetings should last no longer than 2-3 hours, with approximately 1-2 hours reading prior to each meeting.	
Requirements	Experience of physiotherapy for stroke (as a physiotherapist, educator or manager).<br>Confidence to share views and opinions in a small consensus group.	
Training	Any training or information that you require (e.g. about Cochrane systematic reviews) will be provided in the pre-meeting documents or at the meetings.	
Benefits	Opportunity to help shape future physiotherapy stroke services.<br>Use experiences to improve standards of care for stroke patients.<br>Learn new skills and / or make use of existing knowledge and skills.<br>Significant overlap with a number of core dimensions outlined in the Knowledge and Skills Framework (KSF)[2]	
Reimbursement	Any reasonable travel expenses will be reimbursed.	
Contact	Dr Alex Pollock, Research Fellow<br>Nursing Midwifery and Allied Health Professions (NMAHP) Research Unit<br>Glasgow Caledonian University<br>Cowcaddens Rd, Glasgow G4 0BA<br>Phone. 0141 3318100<br>Email alex.pollock@gcu.ac.uk	Dr Pauline Campbell, Researcher<br>Nursing Midwifery and Allied Health Professions (NMAHP) Research Unit<br>Glasgow Caledonian University<br>Cowcaddens Rd, Glasgow G4 0BA<br>Phone. 0141 2731934<br>Email pauline.campbell@gcu.ac.uk

[2] C1 – Communication; C2 – Person and People Development, C4 – Service Improvement; C5 – Quality; HWB6 – Assessment and Treatment Planning; HWB7 – Intervention and Treatments; IK2 – Information Collection and Analysis; IK3 – Knowledge and Information Resources; G1 – Learning and Development and G2 – Development and Innovation
