# Supplementary material for: User involvement in a Cochrane systematic review: using structured methods to enhance the clinical relevance, usefulness and usability of a systematic review update
Source: Syst Rev. 2015 Apr 20;4:55. doi: 10.1186/s13643-015-0023-5 (PMC4407304; doi:10.1186/s13643-015-0023-5)
Supplement: Additional file 2: — Table summarising content and format of stakeholder group meetings. This table provides details of each of the three stakeholder group meetings, including the statements which were discussed and voted on. [file 13643_2015_23_MOESM2_ESM.docx]

**Table summarising content and format of Stakeholder Group meetings.**

|  | **Meeting 1** | **Meeting 2** | **Meeting 3** |
| --- | --- | --- | --- |
| **Time in project (number of months into 12 month project)** | 3.5 months | 5 months | 11 months |
| **Aim of meeting** | To discuss categorisation of interventions and inclusion of evidence from the international trials identified in the 2007 review. | To explore descriptions of treatment components and reach consensus over descriptions and categorisations. | To agree key clinical implications arising from completed review.  To agree dissemination strategies. |
| **Pre-meeting preparation** | Group members sent and asked to read and consider:   - Summary of background to project - Lay summary of 2007 Cochrane review + link to full review - Translations of descriptions of interventions of foreign-language papers which had been ‘awaiting assessment’ in 2007 version | Group members sent descriptions of treatment components from 30 trials (all trials included in 2007 version + papers ‘awaiting assessment’). Asked to complete 2 forms based around categorisation of the interventions within these 30 trials, and how the treatment components might be grouped together within a taxonomy. Group members sent responses in advance of meeting. | None |
| **Presentation of material at meeting** | - What is a Cochrane review? - Overview of 2007 Cochrane review - Details of categorisation of interventions in 2007 version - Exploration of content of foreign-language paper interventions (those ‘awaiting assessment’ in 2007 version) | - Details of published taxonomies of rehabilitation interventions - Summary of responses (anonymous) from group members | - Results of the review - Results of meta-analyses - Results of sub-group analyses - Limitations of analyses |
| **Discussion at meeting** | Discussion was focussed on each statement in turn.  Discussion also led to identification of subgroups which the group felt should be explored within subgroup analyses. | Initial discussion was around advantages and disadvantages of different terminology and taxonomies.  Subsequent discussion was specifically focused on defining and categorising treatment components. | There was discussion around the perceived clinical implications of the findings of each analysis and sub-group analysis. The group members debated the clinical implications and reached agreement on wording of key statements. |
| **Statements discussed and voted on** | **A:** *“The current categories [based on Western classifications] are appropriate and clinical relevant.”*  **B:** *“These international trials [which do not fit into out Western classifications] should be included in our review of physiotherapy treatment approaches.”*  **C**: “*The interventions studied in these [international trials, awaiting assessment] are similar to one another”* | **A***: “The new categories are appropriate and clinically relevant”*  **B:** *“The stated names are appropriate and clinically relevant.”* | No voting was carried out during meeting 3. An evaluation form was completed. |
