# Supplementary material for: User involvement in a Cochrane systematic review: using structured methods to enhance the clinical relevance, usefulness and usability of a systematic review update
Source: Syst Rev. 2015 Apr 20;4:55. doi: 10.1186/s13643-015-0023-5 (PMC4407304; doi:10.1186/s13643-015-0023-5)
Supplement: Additional file 3: — Sample voting slip. This is a copy of one of the voting slips used during stakeholder group meeting 1. The slips were printed on A5 paper. Participants were instructed to circle the appropriate number to show their agreement with the statement and write the reason for their selection in the comments box. [file 13643_2015_23_MOESM3_ESM.docx]

| **A. The current categories are appropriate and clinically relevant.** | | | | |
| --- | --- | --- | --- | --- |
| Strongly agree | Agree | Neither agree or disagree | Disagree | Strongly disagree |
| **1** | **2** | **3** | **4** | **5** |
| ***Any Comments:*** | | | | |
