# Supplementary material for: User involvement in a Cochrane systematic review: using structured methods to enhance the clinical relevance, usefulness and usability of a systematic review update
Source: Syst Rev. 2015 Apr 20;4:55. doi: 10.1186/s13643-015-0023-5 (PMC4407304; doi:10.1186/s13643-015-0023-5)
Supplement: Additional file 4: — Overview of methods used to gain feedback and involvement. A number of different methods were used to communicate with, and gain feedback and involvement of, the stakeholder group members. This table describes the range of methods used, how these were used and the resulting involvement of the stakeholder group members. [file 13643_2015_23_MOESM4_ESM.docx]

**Table providing overview of methods used to gain feedback and involvement of Stakeholder Group members.**

| **Method** | **Description** | **How were these used?** | | **What involvement resulted?** | |
| --- | --- | --- | --- | --- | --- |
| **Personal email conversations** | Emails sent from researcher to individual SG member, possibly in the form of an email conversation. | These were never initiated by the researcher. If a SG member contacted a researcher the researcher replied in a personal email. | | These conversations related to either:   1. A question posed by the SG member in response to a Group email (but reply sent to researcher only). Often this was point of clarification regarding a request for feedback. 2. Practical issues, such as meeting times, travel and parking arrangements, relating to the meetings. | |
| **Group email – provision of information** | Emails sent from researcher to all members of SG group, providing information or a project update, but not specifically requesting feedback. | These were used to:   1. Provide practical details of meetings, such as agenda, venue etc. 2. Suggest background reading (e.g. members were sent link to the published version of the Cochrane review, and translations of descriptions of Chinese physiotherapy interventions prior to attending meeting 1) 3. Provide group members progress updates (e.g. there were 5 months between meetings 2 and 3; during this time a brief update email was sent out) | | These emails elicited few responses. Any responses tended to relate to practical issues. The email sent out made it clear that an email response was not required from members. | |
| **Group email – feedback form** | Emails sent from researcher to all members of SG group, containing a specific request for views or comments relating to a specific query or question, and attaching a structured form for submitting replies. | These were used as a tool to encourage and facilitate individuals to consider a specific problem or issue. Feedback forms were used to encourage thought around   1. How to categorise the interventions delivered within individual studies 2. How to group treatment components together 3. How to define (or name) different groups of treatment components | | Individual feedback was provided, by email by:  a) Only 2 people provided written feedback using the provided form. However all physiotherapist members had attempted the task, and brought thoughts to the subsequent meeting which had been shaped by the exercise.  b) 10 people provided written feedback about how they would group treatment components  c) 8 people suggested names for different categories of treatment components, and 6 people provided additional written considerations around this topic | |
| **Individual email – request for review comments** | Emails sent from researcher to selected members of SG group, requesting specific review and feedback on written materials. | This was used to gain feedback on   1. The Plain Language Summary of the updated Cochrane review. Feedback was requested from a stroke survivor/carer member who had previously expressed interest in this task. 2. A single-page summary of findings, designed for circulation amongst physiotherapists. Feedback was requested from the physiotherapist members only. | | We received   1. Detailed comments and feedback from the individual member on the Plain Language Summary. 2. Brief feedback from 6 physiotherapist members on a draft summary of findings. No one provided detailed review, instead all giving positive confirmation that they were happy with the draft. | |
| **Group email – “vote”** | Email sent by researcher to all members of SG and to all review authors, containing a link to a Doodle poll, and a request to “vote” for preferred choice. | This was used to gain views when Cochrane Stroke Group peer review comments suggested a change to the title of the Cochrane review. Two potential titles were provided and members asked to vote for their preferred title. | | 13 votes were cast within 4 days of the email, proving a quick and efficient way of gaining involvement. There was unanimous support for one title. | |
| **Face-to-face meetings** | (see Additional File 2 for details of the face-to-face meetings) | |  | |  |
| **Group email – request for volunteers for additional involvement** | Email sent by researcher to all SG members asking if they would like to volunteer for additional involvement. | An email asked SG members if they would like to be a co-applicant on a proposal for a symposium session at the Cochrane Symposium (and co-presenter if successful). | | Four SG members responded positively within the given time period. A proposal was drafted by 2 researchers and the 4 volunteer SG members. This was successful. | |
| **Small group teleconference** | One teleconference was held with 2 researchers and 4 SG members, | This teleconference was held specifically to discuss the format of the presentation to be run at the Cochrane Symposium. | | During the teleconference the content of the presentation, and the roles of each of the 6 presenters was agreed. | |
