# Supplementary material for: User involvement in a Cochrane systematic review: using structured methods to enhance the clinical relevance, usefulness and usability of a systematic review update
Source: Syst Rev. 2015 Apr 20;4:55. doi: 10.1186/s13643-015-0023-5 (PMC4407304; doi:10.1186/s13643-015-0023-5)
Supplement: Additional file 5: — Table of individual treatment components, definitions and categories, as defined by the stakeholder group. This table lists the categories and treatment components as defined by the stakeholder group. These categories and treatment components were used to categorise the interventions of all the trials included within the Cochrane review. [file 13643_2015_23_MOESM5_ESM.docx]

**Table:** Individual treatment components, definitions & categories, as defined by the Stakeholder Group

| **Categories** | **Treatment component** | **Description of individual treatment component** |
| --- | --- | --- |
| Assistive devices (AD) | Walking aids | Devices to assist walking, including sticks and frames |
|  | Orthoses for walking | Externally applied orthoses to assist walking, including AFO, knee braces |
|  | Resting splints | Externally applied orthoses to maintain or improve limb alignment |
| Cardiopulmonary interventions (CI) | Aerobic/fitness/endurance training | Activities to improve cardiopulmonary fitness |
| Functional task training (FTT) | ADL training | Practice of tasks relevant to daily life, including both part and whole task practice |
|  | Sitting &/or standing balance training | Various activities performed sitting &/or standing with the aim of improving the ability to balance safely and independently |
|  | Sit-to-stand practice | Practice of tasks aimed at improving ability to stand up and sit down safely and independently |
|  | Transfer practice | Practice of tasks aimed at improving ability to move from one position to another |
|  | Walking | Practice of tasks aimed at improving ambulation |
|  | Stair climbing | Practice of tasks aimed at ability to go up and down stairs |
|  | Upper limb function training | Practice of tasks aimed at improving the ability to move and use the arm, such as reach, grasp, and hand-to-mouth activities |
|  | Described as "MRP" (MRP – Motor Relearning Programme) | Described as MRP |
| Modality (Mo) | Acupuncture | as an adjunct, delivered for either pain relief or movement therapy |
|  | Physical agents (including hot, cold, TENS – Transcutaneous electrical nerve stimulation) | as an adjunct, delivered for either pain relief or movement therapy |
| Musculoskeletal intervention (active) | Muscle strengthening | Practice of activities to progressively increase the ability to generate muscle force, including using body weight and external resistance |
|  | Active & active-assisted movement | Moving a limb through its range of movement, under the patient’s active control with or without assistance |
| Musculoskeletal intervention (passive) | Increasing angle of upright sitting | a form of positioning, to promote early sitting |
|  | Tilt table | To promote early lower limb loading |
|  | Passive movement | Moving a limb through it’s range of movement, whilst the patient is passive |
|  | Body & limb positioning | placing a limb or body part in a supported position, to maintain optimal alignment |
|  | Massage | Manipulation of soft tissue, using the hands or a tool designed for the purpose |
| Neurophysiological intervention | Hands on facilitation of ('normal') movement (Bobath) | Intervention which is described as facilitation of movement, referenced to Bobath or Davies |
|  | Inhibition of abnormal muscle tone / normalising tone (Bobath) | Intervention which is described as inhibition of abnormal muscle tone or as normalising muscle tone, referenced to Bobath or Davies |
|  | Described as "Bobath" | Described as Bobath |
|  | Trunk mobilisations / postural reactions (Bobath) | Intervention which is described as trunk mobilisations or postural reactions to perturbations, referenced to Bobath or Davies |
|  | Proprioceptive Neuromuscular facilitation (PNF – proprioceptive neuromuscular facilitation) | Described as PNF |
|  | Sensorimotor facilitation | The use of excitatory techniques, such as brushing, striking, tapping, icing, to improve sensory awareness and promote muscle activity |
