# Supplementary material for: User involvement in a Cochrane systematic review: using structured methods to enhance the clinical relevance, usefulness and usability of a systematic review update
Source: Syst Rev. 2015 Apr 20;4:55. doi: 10.1186/s13643-015-0023-5 (PMC4407304; doi:10.1186/s13643-015-0023-5)
Supplement: Additional file 6: — Results of written evaluation collected after the third stakeholder group meeting. Written evaluation was collected from nine participants after the third stakeholder group meeting. Participants ranked their agreement with statements 1 to 8 on a five-point scale from strongly agree to strongly disagree. The first table illustrates the agreement assigned by the nine participants. The feedback form also contained four open-questions, and participants provided written comments in response to these. The second table lists all the written responses (unedited and anonymous) from the nine participants to these four open-questions. [file 13643_2015_23_MOESM6_ESM.docx]

**TABLE: WRITTEN EVALUATION FROM STAKEHOLDER GROUP, COLLECTED AFTER 3^RD^ STAKEHOLDER GROUP MEETING**

| **Statement** | **Agreement** |
| --- | --- |
| 1. "I have enjoyed taking part in the SPRUCE stakeholder group" | 9/9 strongly agree |
| 1. "I feel that my involvement has helped make the review more clinically relevant" | 5/9 strongly agree;  4/9 agree |
| 1. "I feel that the views of the stakeholder group did not have any impact on the review update" | 9/9 strongly disagree |
| 1. "I do not feel that the format of the review has not benefited from the involvement of the stakeholder group" | 9/9 strongly disagree |
| 1. "I feel that the review is clinically relevant" | 8/9 strongly agree;  1/9 agree |
| 1. "I feel that the review has implications for my own clinical practice/treatment" | 6/9 strongly agree;  3/9 agree |
| 1. "I will not ensure that my colleagues know about the results of this review" | 9/9 strongly disagree |
| 1. "I think that other Cochrane reviews would benefit from the involvement of a similar stakeholder group" | 9/9 strongly agree |

| **QUESTION** | **FEEDBACK (as written on feedback form by individual respondents)** | | | |
| --- | --- | --- | --- | --- |
| WHAT WAS GOOD ABOUT THE STAKEHOLDER GROUP? | |  | | |
|  | - Broad representative of clinicians - Gaining insight and knowledge of the research process - Being able to give clinical viewpoint, therefore increasing the likelihood of having positive impact on clinical practice - Integration of research/clinicians/patients - appreciation of all perspectives and powerful ness of group opinion/consensus - I feel that the opinions of the stakeholder group were greatly valued. - Good involvement of a mixed group of patients/carers/clinicians - Links clinicians with researchers, allowing research to be more clinically relevant - Clinicians more aware of recent research and processes - Focused yet relaxed, felt opinions were valued - As a carer for a stroke patient I felt I was really listened to - All contributions valued and had influence - Inclusion of views from end users (patients, carers, professionals) - Good open discussion, interesting to hear views on practice | | | |
| WHAT WAS NOT SO GOOD ABOUT THE STAKEHOLDER GROUP? | |  | | |
|  | - Midweek meetings - Would benefit from other representatives e.g. People who would be able to change physio practice- broader MDT- rehab consultants etc. - All good! - Nothing - Nil - I seemed to be on my own: it would have been good to have other patient/carer comments - Travel! - Would be great to have all members of the stakeholder group turn up for all meetings | | | |
| ANY COMMENTS ABOUT THE REVIEW? | | | |  |
|  | - Found out about the review by chance from colleague - Awareness of opportunities to participate would be beneficial - Really interesting - both process of review and clinical implications - Demonstrated effectiveness of treatment approaches but not single specific approaches - Generally a very useful project to be involved in as I feel that the results gained will be widely accessible and relevant - I feel confident about the implications for my clinical practice I.e. Using an eclectic individualised approach. - Also confident in teaching implications to colleagues and students - Seems to have been done very professionally - It has become more clinically user-friendly and applicable - Nominal group process is very well executed | | | |
| ANY OTHER FEEDBACK | | |  | |
|  | - Very helpful for my learning - Really well organised, structured and productive - Very valuable in informing clinical practice - Many thanks for requesting our involvement - All meetings have been very productive, it has also been very useful listening to the views and opinions of more experienced clinicians and very thought provoking - Thank you - On with the good work! Other Cochrane groups please copy - Contribution to publication? - Hope this methodology catches on! - Having more opinions on analysis of info etc is helpful when thinking of recommendations and conclusions etc | | | |
